# Reanalysis of the Effect of Vitamin C on Mortality in the CITRIS-ALI Trial: Important Findings Dismissed in the Trial Report

**DOI:** 10.3389/fmed.2020.590853

**Published:** 2020-10-07

**Authors:** Harri Hemilä, Elizabeth Chalker

**Affiliations:** ^1^Department of Public Health, University of Helsinki, Helsinki, Finland; ^2^University of Sydney, Sydney, NSW, Australia

**Keywords:** critical care, intensive care units, randomized controlled trial, mortality, sepsis, survival analysis, time factors, antioxidants

## Introduction

The CITRIS-ALI trial was a randomized clinical trial assessing the effect of vitamin C on organ failure and biomarkers of inflammation and vascular injury in patients with sepsis and severe acute respiratory failure (1). Patients with sepsis and acute respiratory distress syndrome were administered 50 mg/kg vitamin C intravenously for four days. The trial report states that, “*The Kaplan-Meier survival curves for the 2 groups were significantly different by the Wilcoxon test (*$\chi_{\left[1df\right]}^{2}$ = *6.5; P* = *0.01)*” (1). However, this finding was not mentioned in the abstract and it was downplayed in the discussion section.

Previously, we conducted a meta-analysis of 12 controlled trials assessing the effect of vitamin C on intensive care unit (ICU) length of stay. The meta-analysis included 1,766 patients and found that vitamin C shortened ICU stay on average by 8% (95% CI: 4-11%) (2). One of the included trials found that vitamin C reduced mortality in sepsis patients by 78% (*P* = 0.01) (3). Both our meta-analysis and this trial are relevant when considering the effect of vitamin C on ICU patients, but neither was cited in the CITRIS-ALI trial report (1).

The CITRIS-ALI trial report suggested that the effect of vitamin C on mortality might be explained by multiple comparisons (1). The purpose of this reanalysis is to argue that the multiple comparisons issue is an unlikely explanation for the published survival findings.

## Reanalysis of the CITRIS Trial

In the CITRIS-ALI trial registration, mortality was listed as one of 18 secondary outcomes (4). Most of them are biomarkers with little clinical importance. Only four secondary outcomes are unambiguously clinically relevant: ventilator-free days, ICU-free days, hospital-free days, and mortality. The effect of vitamin C was statistically significant for three of them: ICU-free days, hospital-free days, and mortality (1). Furthermore, none of the secondary or primary outcomes matches mortality in terms of clinical importance.

In the discussion section (1), Fowler et al. write that “*Among the 46 secondary outcomes that were examined in this trial, 43 showed no significant differences between the vitamin C group and the placebo group, although vitamin C compared with placebo was associated with a significant reduction in 28-day all-cause mortality, and with significantly increased ICU-free days to day 28 and hospital-free days to day 60. However, these findings were based on analyses that did not account for multiple comparisons and therefore must be considered exploratory*.” This comment misleads the readers.

First, the trial registration listed just 18 secondary outcomes (4). Although many of them were measured at several time points, this should not equate to 46 independent secondary outcomes. If vitamin C has no effect on a biomarker, it is likely that the lack of effect is uniform over time. Counting the measurements at several time points as if they were independent makes the multiple comparison issue appear much greater than it is.

Second, a researcher interested in the effects of vitamin C on mortality can legitimately deny interest in the secondary outcomes that are just biomarkers such as the levels of platelets, bilirubin, procalcitonin, tissue factor pathway inhibitor, receptor for advanced glycation endpoints, and so on (4). The use of biomarkers has been popular since measuring biomarkers/surrogates is much quicker and much less expensive than measuring clinically relevant outcomes. However, the wide-spread use of surrogates has been severely criticized. There are numerous examples that demonstrate that the effects on surrogate endpoints can diverge from the effects on clinically relevant outcomes (5–8). However, four of the measured outcomes in the CITRIS-ALI trial were clinically relevant. When there is a significant difference between the treatment groups in three out of four of the clinically relevant outcomes, and a significant effect for the most important outcome, mortality (1), the multiple comparisons issue is not a valid explanation for the findings. The measurement of many biomarkers should not be used to dismiss the findings on the clinically relevant outcomes. Although multiple comparisons are a concern when researchers calculate numerous associations without justified research questions, the issue has often been greatly exaggerated (9–11).

Third, prior learning is relevant for determining research questions and interpreting findings. Given that in earlier studies vitamin C was found to shorten ICU and hospital stay (2, 12), and decrease mortality (3), research on these clinically relevant outcomes is not a fishing expedition without biological rationale, but testing well-justified hypotheses. This previous research on vitamin C and the three clinically relevant outcomes was not considered by Fowler et al. (1).

In addition to exaggerating the multiple comparisons problem, the analysis of mortality findings was not optimal in the CITRIS-ALI trial. Vitamin C was administered for four days, but mortality was analyzed over 28 days. Thus, if vitamin C had an effect only around the time of administration, extension of the mortality analysis by 24 days in the absence of vitamin C may camouflage any potential benefits. Inspection of the survival curves (1) indicates that the vitamin C and placebo groups differed in the early follow-up, but not in the late follow-up. These differences can be tested statistically.

When separate vitamin C effects in the early and late follow-up periods are accounted for, the improvement in the Cox regression model can be calculated by the likelihood ratio test. Table 1 shows that adding a separate early period vitamin C effect for four days leads to the greatest improvement in the Cox regression model.

**Table 1:** Improvement in the Cox regression model by allowing different vitamin C effects for two consecutive time periods.

**Periods of vitamin C effect (days)**	Improvement*	
	**$\chi_{\left(1\text{df}\right)}^{2}$**	**P**
0-28	Reference	
0-1 and 2-28	4.54	0.033
0-2 and 3-28	4.82	0.028
0-3 and 4-28	6.52	0.011
0-4 and 5-28	7.65	0.006
0-5 and 6-28	7.22	0.007
0-6 and 7-28	3.49	0.061
0-7 and 8-28	1.41	0.236

* *The improvement in the Cox regression model over the reference model was tested by the likelihood ratio test. The reference model estimates that vitamin C decreases mortality uniformly by RR = 0.57 (95% CI: 0.34-0.95; P = 0.028). Compared with the model with no vitamin C effect, the best-fitting two-period model (0–4 days and 5–28 days) improves the Cox regression with $\chi_{\left(2df\right)}^{\text{2}}$ = 12.5 (P = 0.002). See the Supplementary File for the extraction of mortality data from Figure 3 of the CITRIS-ALI trial report (1) and the calculations for this table*.

The best-fitting model in Table 1 indicates that vitamin C decreased mortality in the period up to day 4 with RR = 0.19 (95% CI 0.06-0.55), but had no effect from day 5 onwards with RR = 0.95 (95% CI 0.50-1.79). Compared to the model with no vitamin C effect over the 28-day period, this two-period model improves the Cox regression with $\chi_{\left(2\text{df}\right)}^{2}$ = 12.5 (*P* = 0.002). This strong evidence of within-follow-up heterogeneity in the vitamin C effect further challenges the suggestion that the effect of vitamin C on mortality may have been an artifact explained by multiple comparisons (1). If the overall difference between the vitamin C and placebo groups is assumed to be caused purely by random variation, we would not expect to observe such strong evidence of systematic differences between the early and late follow-up periods.

Furthermore, given that vitamin C was administered for only four days, it is reasonable to examine the effect of vitamin C on mortality over the same period. By the end of day 4, the mortality rate was 22.9% (19/83) in the placebo group and 4.8% (4/84) in the vitamin C group, with *P* = 0.0007. This difference of 18.1 percentage points corresponds to the number needed to treat equal to 5.5 (95% CI: 3.5–12.5).

Fowler et al. calculated *P* = 0.01 for the difference between the survival curves for the vitamin C and placebo groups (1). However, as shown above, the evidence for vitamin C is much stronger if the analysis is restricted to the four days during which vitamin C was administered, or if two distinct periods for the effect of vitamin C are accounted for.

There is evidence that the effects of vitamin C may be greatest for ICU patients who are the most ill (2, 13). It is likely that patients in the CITRIS-ALI trial who were alive a week after the start of treatment were less ill at baseline compared with those who died within four days. Thus, the evidence for greater benefit for the most ill patients also motivates a separate analysis over the early part of the follow-up.

There are numerous potential biochemical explanations for the effects of vitamin C on severely ill patients (2, 14–16). It participates in the synthesis of norepinephrine, carnitine, nitric oxide, and several neuropeptides. It also participates in the demethylation of DNA and histones and thereby influences the epigenome; vitamin C may demethylate over 1,000 genes in embryonic stem cells. Vitamin C also hydroxylates specific proline residues in hypoxia-inducible factor-I, which is a transcription factor involved in oxygen sensing, and thereby may regulate several hundred genes. Finally, as a major antioxidant vitamin C can have a wide range of nonspecific effects.

## Conclusions

The CITRIS-ALI trial findings indicate that the effect of vitamin C on mortality is not equal to placebo. This is an important finding which should not be dismissed solely because of the multiple comparisons issue. It is noteworthy that the 81% decrease in mortality during vitamin C administration in the CITRIS-ALI trial is similar to the previously reported 78% decrease in mortality (3). Nevertheless, it is unlikely that this estimate can be widely generalized because there is evidence indicating that the effect of vitamin C on ICU patients depends substantially on the illness-severity of patients (13). Further research is required to investigate this relationship.

## Author Contributions

HH planned the study, measured the published survival curve (1), entered the data into a spreadsheet and carried out the statistical analysis, and wrote the draft manuscript. EC checked that the entered data were consistent with the published survival curve and participated in the critical revision of the manuscript. Both authors read and approved the final manuscript.

## Conflict of Interest

The authors declare that the research was conducted in the absence of any commercial or financial relationships that could be construed as a potential conflict of interest.
