# Supplementary material for: Reanalysis of the Effect of Vitamin C on Mortality in the CITRIS-ALI Trial: Important Findings Dismissed in the Trial Report
Source: Front Med (Lausanne). 2020 Oct 7;7:590853. doi: 10.3389/fmed.2020.590853 (PMC7575729; doi:10.3389/fmed.2020.590853)
Supplement: Supplementary file 1 [file Data_Sheet_1.PDF]

# Reanalysis of the effect of vitamin C on mortality in the CITRIS-ALI trial

Harri Hemilä 1) and Elizabeth Chalker 2)

1. Department of Public Health, University of Helsinki, POB 41, FI 00014, FINLAND
2. University of Sydney, Sydney, Australia

Harri Hemilä
http://orcid.org/0000-0002-4710-307X
https://www.mv.helsinki.fi/home/hemila/
harri.hemila@helsinki.fi

version 2020-8-18

Supplement to a manuscript submitted to
https://www.frontiersin.org/journals/medicine/sections/intensive-care-medicine-and-anesthesiology#

This supplement describes the extraction of mortality data from Figure 3 of the CITRIS-ALI trial report and the calculations for Cox regression.

The CITRIS-ALI trial report was published in:

> Fowler AA, Truwit JD, Hite RD, Morris PE, DeWilde C, Priday A, et al.
> Effect of vitamin C infusion on organ failure and biomarkers of inflammation and vascular injury in patients with sepsis and severe acute respiratory failure: the CITRIS-ALI randomized clinical trial. JAMA 2019;322:1261–1270.
> https://doi.org/10.1001/jama.2019.11825

**Contents** **page**

Extraction of mortality data 2
Cox regression: two time periods of vitamin C effects 4
Calculation of the NNT for the 4-day duration of vitamin C supplementation 9
Cox regression over first four days:
the effect of vitamin C during the actual vitamin C administration 10
Risk ratio and Fisher exact test over the first four days 11

Deaths in the CITRIS-ALI trial were shown as steps in Figure 3 in the CITRIS-ALI report. The figure is copied below to illustrate the measurement of deaths over the follow-up period.

When the number of participants is quite low, it is possible to back-calculate from the survival curve the number of participants (deaths) on each step. The size of the steps was measured from the digital figure as pixels and the scale of the figure as pixels was used to determine the number of deaths on each step. A spreadsheet was used to transform the pixel-values to the mortality scale on the Y-axis from 0% to 60%. The extraction of step sizes and the calculations on the spread sheet are shown on page 3 (Table 1).

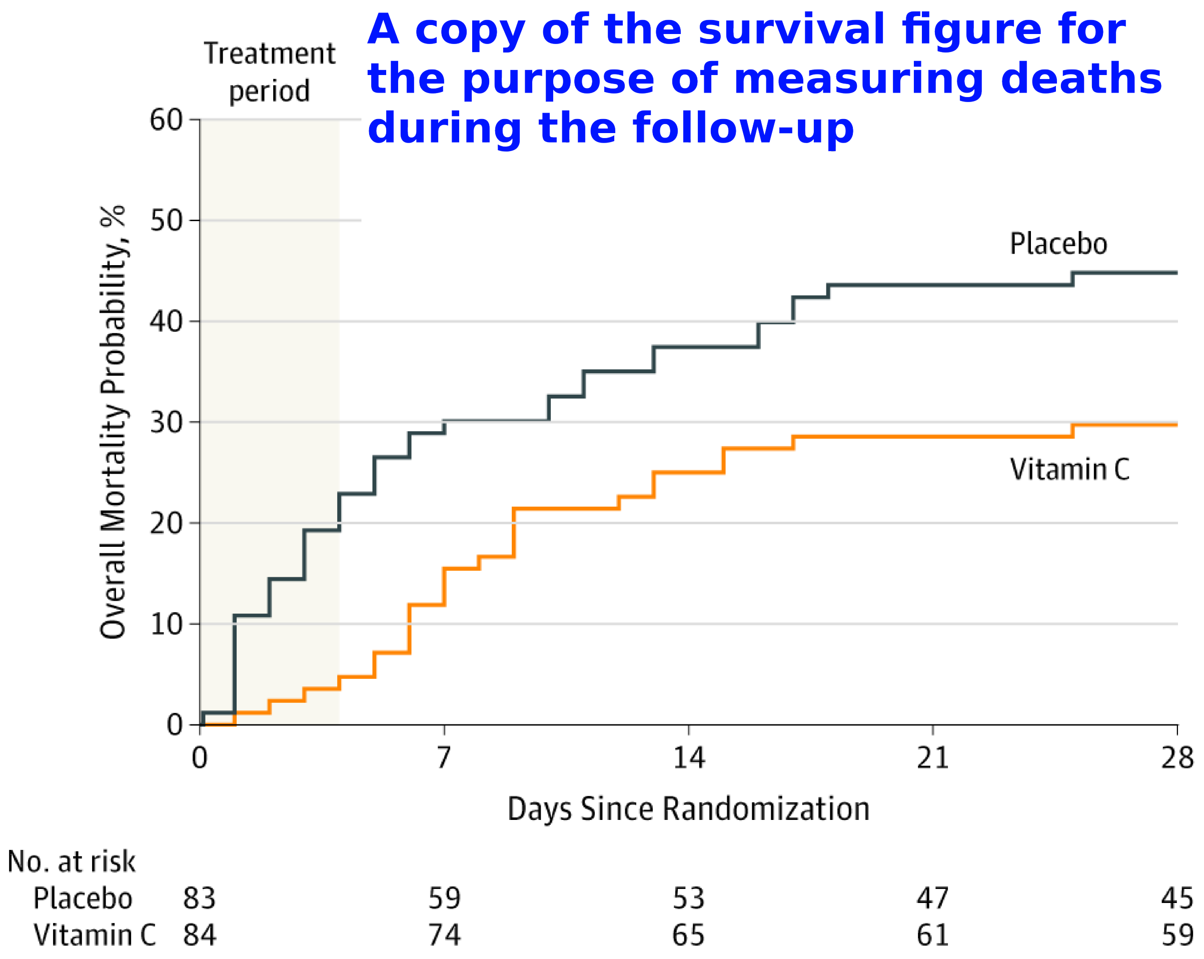

**Table 1: All-cause mortality from randomization (Day 0) to Day 28 among patients with sepsis associated ARDS (from Fowler et al. Figure 3)**

The left-hand side of this table shows the measurement of the steps in Figure 3.
The right-hand side shows the number of deaths by the end of the given day.
The number of patients (deaths) on each step could be inferred with great accuracy.

| | | | | | | | | |
|---|---|---|---|---|---|---|---|---|
| Scale | | | | Vitamin C | Placebo | | | |
| % mortality | pixels | pixels | N: | 84 | 83 | | | |
| 60 | 462 | | | | | | | |
| 0 | 2664 | | | | | Number of patients on each step Calculated | | |
| | | | | N cumulative Calculated | | | | |
| | | | | | | Vitamin C | Placebo | |
| Day | Vitamin C | Placebo | | Vitamin C | Placebo | vitC | Placebo | Death |
| 0 | 2664 | 2664 | | 0.00 | 0.00 | 0.00 | 0.00 | 1 |
| 1 | 2619 | 2266 | | 1.03 | 9.00 | 1.03 | 9.00 | 1 |
| 2 | 2577 | 2134 | | 1.99 | 11.99 | 0.96 | 2.99 | 1 |
| 3 | 2534 | 1957 | | 2.98 | 15.99 | 0.98 | 4.00 | 1 |
| 4 | 2490 | 1824 | | 3.98 | 19.00 | 1.01 | 3.01 | 1 |
| 5 | 2401 | 1692 | | 6.02 | 21.98 | 2.04 | 2.99 | 1 |
| 6 | 2227 | 1604 | | 10.00 | 23.97 | 3.98 | 1.99 | 1 |
| 7 | 2096 | 1559 | | 13.00 | 24.99 | 3.00 | 1.02 | 1 |
| 8 | 2053 | 1559 | | 13.98 | 24.99 | 0.98 | 0.00 | 1 |
| 9 | 1877 | 1559 | | 18.01 | 24.99 | 4.03 | 0.00 | 1 |
| 10 | 1877 | 1470 | | 18.01 | 27.00 | 0.00 | 2.01 | 1 |
| 11 | 1877 | 1379 | | 18.01 | 29.06 | 0.00 | 2.06 | 1 |
| 12 | 1835 | 1379 | | 18.97 | 29.06 | 0.96 | 0.00 | 1 |
| 13 | 1746 | 1290 | | 21.01 | 31.07 | 2.04 | 2.01 | 1 |
| 14 | 1746 | 1290 | | 21.01 | 31.07 | 0.00 | 0.00 | 1 |
| 15 | 1660 | 1290 | | 22.98 | 31.07 | 1.97 | 0.00 | 1 |
| 16 | 1660 | 1199 | | 22.98 | 33.13 | 0.00 | 2.06 | 1 |
| 17 | 1616 | 1109 | | 23.99 | 35.17 | 1.01 | 2.04 | 1 |
| 18 | 1616 | 1065 | | 23.99 | 36.16 | 0.00 | 1.00 | 1 |
| 19 | 1616 | 1065 | | 23.99 | 36.16 | 0.00 | 0.00 | 1 |
| 20 | 1616 | 1065 | | 23.99 | 36.16 | 0.00 | 0.00 | 1 |
| 21 | 1616 | 1065 | | 23.99 | 36.16 | 0.00 | 0.00 | 1 |
| 22 | 1616 | 1065 | | 23.99 | 36.16 | 0.00 | 0.00 | 1 |
| 23 | 1616 | 1065 | | 23.99 | 36.16 | 0.00 | 0.00 | 1 |
| 24 | 1616 | 1065 | | 23.99 | 36.16 | 0.00 | 0.00 | 1 |
| 25 | 1573 | 1020 | | 24.97 | 37.18 | 0.98 | 1.02 | 1 |
| 26 | 1573 | 1020 | | 24.97 | 37.18 | 0.00 | 0.00 | 1 |
| 27 | 1573 | 1020 | | 24.97 | 37.18 | 0.00 | 0.00 | 1 |
| 28 | 1573 | 1020 | | 24.97 | 37.18 | 0.00 | 0.00 | 1 |
| | | | | Alive at the end of day 28: | | 59.03 | 45.82 | 0 |
| | | | | | Total deaths: | 25.0 | 37.2 | |

## Cox regression: two time periods of vitamin C effects

Printouts of the calculation of the Cox regression models.

The **base** model indicates model with only *one uniform vitamin C effect* over the 28-day follow-up.

```
> CITRIX_S <- Surv(CITRIS$Day, CITRIS$Censored)
> CITRIX_S
  [1]  1   1   1   1   1   1   1   1   1   2   2   2   3   3   3   3   4   4   4   5   5   5   6   6   7  10
 [27] 10  11  11  13  13  16  16  17  17  18  25  28+ 28+ 28+ 28+ 28+ 28+ 28+ 28+ 28+ 28+ 28+ 28+ 28+ 28+ 28+
 [53] 28+ 28+ 28+ 28+ 28+ 28+ 28+ 28+ 28+ 28+ 28+ 28+ 28+ 28+ 28+ 28+ 28+ 28+ 28+ 28+ 28+ 28+ 28+ 28+ 28+ 28+
 [79] 28+ 28+ 28+ 28+ 28+  1   2   3   4   5   5   6   6   6   6   7   7   7   8   9   9   9   9  12  13  13
[105] 15  15  17  25  28+ 28+ 28+ 28+ 28+ 28+ 28+ 28+ 28+ 28+ 28+ 28+ 28+ 28+ 28+ 28+ 28+ 28+ 28+ 28+ 28+ 28+
[131] 28+ 28+ 28+ 28+ 28+ 28+ 28+ 28+ 28+ 28+ 28+ 28+ 28+ 28+ 28+ 28+ 28+ 28+ 28+ 28+ 28+ 28+ 28+ 28+ 28+ 28+
[157] 28+ 28+ 28+ 28+ 28+ 28+ 28+ 28+ 28+ 28+ 28+
>
> 
> base   <- coxph(CITRIX_S ~ CITRIS$VitC, method = "efron")
> summary(base)
Call:
coxph(formula = CITRIX_S ~ CITRIS$VitC, method = "efron")

  n= 167, number of events= 62

               coef exp(coef) se(coef)      z Pr(>|z|)
CITRIS$VitC -0.5639    0.5690   0.2590 -2.177   0.0295 *
---


            exp(coef) exp(-coef) lower .95 upper .95
CITRIS$VitC     0.569      1.758    0.3425    0.9453

Concordance= 0.579  (se = 0.031 )
Likelihood ratio test= 4.85  on 1 df,   p=0.03
Wald test            = 4.74  on 1 df,   p=0.03
Score (logrank) test = 4.87  on 1 df,   p=0.03

> lrtest(base)
Likelihood ratio test

Model 1: CITRIX_S ~ CITRIS$VitC
Model 2: CITRIX_S ~ 1
  #Df  LogLik Df  Chisq Pr(>Chisq)
1   1 -301.84
2   0 -304.27 -1 4.8528     0.0276 *
---
```

**Adding a second vitamin C effect time period.** The first lines of the code divide the follow-up between days 1 and 2, ie. the first period is from the beginning to the end of day 1 and second is from day 2 to day 28. The next lines divide the follow-up between days 2 and 3, and so on.

```
> days1 <- survSplit(Surv(CITRIS$Day, CITRIS$Censored) ~ ., cut=c(1.5),
episode= "tgroup", data =CITRIS)
> days1
> days1b <- coxph(Surv(tstart, tstop, event) ~ VitC:strata(tgroup), data=days1,
method = "efron")
> days1b
Call:
coxph(formula = Surv(tstart, tstop, event) ~ VitC:strata(tgroup),
    data = days1, method = "efron")

                               coef exp(coef) se(coef)      z      p
VitC:strata(tgroup)tgroup=1 -2.2547    0.1049   1.0541 -2.139 0.0324
VitC:strata(tgroup)tgroup=2 -0.3477    0.7063   0.2782 -1.250 0.2114

Likelihood ratio test=9.4  on 2 df, p=0.009113
n= 324, number of events= 62
> lrtest(base,days1b)


Likelihood ratio test

Model 1: CITRIX_S ~ CITRIS$VitC
Model 2: Surv(tstart, tstop, event) ~ VitC:strata(tgroup)
  #Df  LogLik Df  Chisq Pr(>Chisq)
1   1 -301.84
2   2 -299.57  1 4.5432     0.033
---


> days2 <- survSplit(Surv(CITRIS$Day, CITRIS$Censored) ~ ., , cut=c(2.5),
episode= "tgroup", data =CITRIS)
> days2b <- coxph(Surv(tstart, tstop, event) ~ VitC:strata(tgroup), data=days2,
method = "efron")
> days2b
Call:
coxph(formula = Surv(tstart, tstop, event) ~ VitC:strata(tgroup),
    data = days2, method = "efron")

                               coef exp(coef) se(coef)      z      p
VitC:strata(tgroup)tgroup=1 -1.8690    0.1543   0.7638 -2.447 0.0144
VitC:strata(tgroup)tgroup=2 -0.2826    0.7539   0.2890 -0.978 0.3282

Likelihood ratio test=9.67  on 2 df, p=0.007938
n= 320, number of events= 62
> lrtest(base,days2b)
Likelihood ratio test

Model 1: CITRIX_S ~ CITRIS$VitC
Model 2: Surv(tstart, tstop, event) ~ VitC:strata(tgroup)
  #Df  LogLik Df  Chisq Pr(>Chisq)
1   1 -301.84
2   2 -299.44  1 4.8194     0.028
```

```
> days3 <- survSplit(Surv(CITRIS$Day, CITRIS$Censored) ~ ., cut=c(3.5),
episode= "tgroup", data =CITRIS)
> days3b <- coxph(Surv(tstart, tstop, event) ~ VitC:strata(tgroup), data=days3,
method = "efron")
> days3b
Call:
coxph(formula = Surv(tstart, tstop, event) ~ VitC:strata(tgroup),
    data = days3, method = "efron")

                                coef exp(coef) se(coef)      z       p
VitC:strata(tgroup)tgroup=1 -1.7741    0.1696   0.6293 -2.819 0.00481
VitC:strata(tgroup)tgroup=2 -0.1569    0.8548   0.3051 -0.514 0.60700

Likelihood ratio test=11.37  on 2 df, p=0.003394
n= 315, number of events= 62
> lrtest(base,days3b)
Likelihood ratio test

Model 1: CITRIX_S ~ CITRIS$VitC
Model 2: Surv(tstart, tstop, event) ~ VitC:strata(tgroup)
  #Df  LogLik Df  Chisq Pr(>Chisq)
1   1 -301.84
2   2 -298.58  1 6.5185    0.0106
---



> days4 <- survSplit(Surv(CITRIS$Day, CITRIS$Censored) ~ ., cut=c(4.5),
episode= "tgroup", data =CITRIS)
> days4b <- coxph(Surv(tstart, tstop, event) ~ VitC:strata(tgroup), data=days4,
method = "efron")
> days4b
Call:
coxph(formula = Surv(tstart, tstop, event) ~ VitC:strata(tgroup),
    data = days4, method = "efron")

                                 coef exp(coef) se(coef)      z       p
VitC:strata(tgroup)tgroup=1 -1.67644   0.18704  0.55027 -3.047 0.00231
VitC:strata(tgroup)tgroup=2 -0.04942   0.95178  0.32125 -0.154 0.87775

Likelihood ratio test=12.5  on 2 df, p=0.00193
n= 311, number of events= 62
> lrtest(base,days4b)
Likelihood ratio test

Model 1: CITRIX_S ~ CITRIS$VitC
Model 2: Surv(tstart, tstop, event) ~ VitC:strata(tgroup)
  #Df  LogLik Df  Chisq Pr(>Chisq)
1   1 -301.84
2   2 -298.02  1 7.6459    0.0056
---
> summary(days4b)
Call:
coxph(formula = Surv(tstart, tstop, event) ~ VitC:strata(tgroup),
    data = days4, method = "efron")

                            exp(coef) exp(-coef) lower .95 upper .95
VitC:strata(tgroup)tgroup=1    0.1870      5.346   0.06361    0.5499
VitC:strata(tgroup)tgroup=2    0.9518      1.051   0.50710    1.7864
```

```
> days5 <- survSplit(Surv(CITRIS$Day, CITRIS$Censored) ~ ., cut=c(5.5),
episode= "tgroup", data =CITRIS)
> days5b <- coxph(Surv(tstart, tstop, event) ~ VitC:strata(tgroup), data=days5,
method = "efron")
> days5b
Call:
coxph(formula = Surv(tstart, tstop, event) ~ VitC:strata(tgroup),
    data = days5, method = "efron")

                                coef exp(coef) se(coef)      z      p
VitC:strata(tgroup)tgroup=1 -1.43833   0.23732  0.46076 -3.122 0.0018
VitC:strata(tgroup)tgroup=2  0.03703   1.03773  0.34544  0.107 0.9146

Likelihood ratio test=12.08  on 2 df, p=0.002385
n= 306, number of events= 62
> lrtest(days5b)
Likelihood ratio test

Model 1: Surv(tstart, tstop, event) ~ VitC:strata(tgroup)
Model 2: Surv(tstart, tstop, event) ~ 1
  #Df  LogLik Df  Chisq Pr(>Chisq)
1   2 -298.23
2   0 -304.27 -2 12.077   0.002385 **
---

> lrtest(base,days5b)
Likelihood ratio test

Model 1: CITRIX_S ~ CITRIS$VitC
Model 2: Surv(tstart, tstop, event) ~ VitC:strata(tgroup)
  #Df  LogLik Df  Chisq Pr(>Chisq)
1   1 -301.84
2   2 -298.23  1 7.2242   0.0071
---


> days6 <- survSplit(Surv(CITRIS$Day, CITRIS$Censored) ~ ., cut=c(6.5),
episode= "tgroup", data =CITRIS)
> days6b <- coxph(Surv(tstart, tstop, event) ~ VitC:strata(tgroup), data=days6,
method = "efron")
> days6b
Call:
coxph(formula = Surv(tstart, tstop, event) ~ VitC:strata(tgroup),
    data = days6, method = "efron")

                                coef exp(coef) se(coef)      z       p
VitC:strata(tgroup)tgroup=1 -1.03195   0.35631  0.37660 -2.740 0.00614
VitC:strata(tgroup)tgroup=2 -0.04803   0.95310  0.37897 -0.127 0.89914

Likelihood ratio test=8.34  on 2 df, p=0.01543
n= 300, number of events= 62
> lrtest(base,days6b)
Likelihood ratio test

Model 1: CITRIX_S ~ CITRIS$VitC
Model 2: Surv(tstart, tstop, event) ~ VitC:strata(tgroup)
  #Df  LogLik Df  Chisq Pr(>Chisq)
1   1 -301.84
2   2 -300.10  1 3.4903     0.061
```

```
> days7 <- survSplit(Surv(CITRIS$Day, CITRIS$Censored) ~ ., cut=c(7.5),
episode= "tgroup", data =CITRIS)
> days7b <- coxph(Surv(tstart, tstop, event) ~ VitC:strata(tgroup), data=days7,
method = "efron")
> days7b
Call:
coxph(formula = Surv(tstart, tstop, event) ~ VitC:strata(tgroup),
    data = days7, method = "efron")

                               coef exp(coef) se(coef)      z     p
VitC:strata(tgroup)tgroup=1 -0.8167    0.4419   0.3421 -2.387 0.017
VitC:strata(tgroup)tgroup=2 -0.1868    0.8296   0.4083 -0.457 0.647

Likelihood ratio test=6.26  on 2 df, p=0.04371
n= 296, number of events= 62
> lrtest(base,days7b)
Likelihood ratio test

Model 1: CITRIX_S ~ CITRIS$VitC
Model 2: Surv(tstart, tstop, event) ~ VitC:strata(tgroup)
  #Df  LogLik Df  Chisq Pr(>Chisq)
1   1 -301.84
2   2 -301.14  1 1.4073      0.23
```

**Calculation of the NNT for the 4-day duration of vitamin C supplementation**

```
> prop.test(c(4,19),c(84,83),correct=FALSE)

        2-sample test for equality of proportions without continuity correction

data:  c(4, 19) out of c(84, 83)
X-squared = 12, df = 1, p-value = 7e-04
alternative hypothesis: two.sided
95 percent confidence interval:
 -0.28251 -0.08009
sample estimates:
 prop 1  prop 2
0.04762 0.22892

>
> (Difference = 0.22892 - 0.04762 )
[1] 0.1813
> (NNT = 1/Difference)
[1] 5.516
>
> # 95% CI for NNT
>
> (1/0.28251)
[1] 3.54
> (1/0.08009)
[1] 12.49
```

**Cox regression over first four days:**
**the effect of vitamin C during the actual vitamin C administration.**

```
CITRIX4Day_S <- Surv(CITRIS4Day$Day, CITRIS4Day$Censored)
> CITRIX4Day_S
  [1]  1   1   1   1   1   1   1   1   1   2   2   2   3   3   3   3   4   4   4    5+  5+  5+  6+  6+  7+ 10+
 [27] 10+ 11+ 11+ 13+ 13+ 16+ 16+ 17+ 17+ 18+ 25+ 28+ 28+ 28+ 28+ 28+ 28+ 28+ 28+ 28+ 28+ 28+ 28+ 28+ 28+ 28+
 [53] 28+ 28+ 28+ 28+ 28+ 28+ 28+ 28+ 28+ 28+ 28+ 28+ 28+ 28+ 28+ 28+ 28+ 28+ 28+ 28+ 28+ 28+ 28+ 28+ 28+ 28+
 [79] 28+ 28+ 28+ 28+ 28+  1   2   3   4    5+  5+  6+  6+  6+  6+  7+  7+  7+  8+  9+  9+  9+  9+ 12+ 13+ 13+
[105] 15+ 15+ 17+ 25+ 28+ 28+ 28+ 28+ 28+ 28+ 28+ 28+ 28+ 28+ 28+ 28+ 28+ 28+ 28+ 28+ 28+ 28+ 28+ 28+ 28+ 28+
[131] 28+ 28+ 28+ 28+ 28+ 28+ 28+ 28+ 28+ 28+ 28+ 28+ 28+ 28+ 28+ 28+ 28+ 28+ 28+ 28+ 28+ 28+ 28+ 28+ 28+ 28+
[157] 28+ 28+ 28+ 28+ 28+ 28+ 28+ 28+ 28+ 28+ 28+
>


>
> Only4D   <- coxph(CITRIX4Day_S ~ CITRIS4Day$VitC, method = "efron")
> summary(Only4D)
Call:
coxph(formula = CITRIX4Day_S ~ CITRIS4Day$VitC, method = "efron")

  n= 167, number of events= 23

                   coef exp(coef) se(coef)      z Pr(>|z|)
CITRIS4Day$VitC -1.6764    0.1870   0.5503 -3.047  0.00231 **
---


                exp(coef) exp(-coef) lower .95 upper .95
CITRIS4Day$VitC     0.187      5.346   0.06361    0.5499

Concordance= 0.683  (se = 0.042 )
Likelihood ratio test= 12.48  on 1 df,   p=4e-04
Wald test            = 9.28  on 1 df,   p=0.002
Score (logrank) test = 11.66  on 1 df,   p=6e-04

> lrtest(Only4D)
Likelihood ratio test

Model 1: CITRIX4Day_S ~ CITRIS4Day$VitC
Model 2: CITRIX4Day_S ~ 1
  #Df  LogLik Df  Chisq Pr(>Chisq)
1   1 -109.89
2   0 -116.13 -1 12.475   0.00041

---
```

**Risk ratio and Fisher exact test over the first four days**

Over the first four days when vitamin C was administered, there were
4 deaths in the vitamin C group of 84 participants
and
19 deaths in the placebo group of 83 participants.

This leads to a risk ratio of RR = 0.21 (95% CI 0.07-0.59):

```
> riskratio(4, 19, 84, 83, conf.level=0.95, p.calc.by.independence=TRUE)
            Disease Nondisease Total
Exposed           4         80    84
Nonexposed       19         64    83

        Risk ratio estimate and its significance probability

data:  4 19 84 83

p-value = 0.00070
95 percent confidence interval:
 0.0739    0.585
sample estimates:
[1] 0.208
```

```
> CITRIS4D <- matrix(c(4, 19, 80, 64),nrow=2)
> CITRIS4D
     [,1] [,2]
[1,]    4   80
[2,]   19   64
> fisher.test(CITRIS4D)

        Fisher's Exact Test for Count Data

data:  CITRIS4D
p-value = 0.00065
alternative hypothesis: true odds ratio is not equal to 1
95 percent confidence interval:
 0.040 0.546
sample estimates:
odds ratio
 0.17
```
